# Elevated IL-6 Receptor Expression on CD4^+ ^T Cells contributes to the increased Th17 Responses in patients with Chronic Hepatitis B

**DOI:** 10.1186/1743-422X-8-270

**Published:** 2011-06-03

**Authors:** Fan Zhang, Simin Yao, Jing Yuan, Mingxia Zhang, Qing He, Guilin Yang, Zhiliang Gao, Hong Liu, Xinchun Chen, Boping Zhou

**Affiliations:** 1 Department of Infectious Diseases, the third affiliated hospital of Sun-Yat-Sen university, Guangzhou, China; 2 Shenzhen Key laboratory for Diagnosis& Treatment of Infectious Diseases, Shenzhen Third People's Hospital, Guangdong Medical College, Shenzhen, China; 3 Shenzhen Nanshan Center for Chronic Disease Control, Shenzhen, China

## Abstract

**Background:**

Increased numbers of Interleukin-17-producing CD4^+ ^T cells (Th17) have been found in association with hepatitis B virus (HBV)-induced liver injury. However, the mechanism underlying the increase of Th17 responses in patients with HBV infection remains unclear. In this study, we investigate the possible regulatory mechanisms of increased Th17 responses in patients with chronic hepatitis B(CHB).

**Methods:**

Th17 response and IL-6R expression on CD4^+ ^T cells in peripheral blood samples were determined by flow cytometry. Cytokines TGF-β, IL-1β, IL-6 and IL-17 in plasma and/or supernatant samples were determined by ELISA and the IL-17 and IL-6R mRNA levels were quantified by quantitative real-time reverse polymerase chain reaction.

**Results:**

All these data indicated that the frequency of periphery Th17 cells is significantly correlated with the percentage of CD4**^+ ^**T cells expressing IL-6R in CHB patients. CD4^+ ^T cells from patients with CHB, but not those from healthy donors, produced higher levels of IL-17 and had more IL-6R expression upon stimulation with the HBV core antigen (HBcAg) in vitro. The PMA/ionomycin and HBcAg -stimulated up-regulation of IL-17 production by CD4^+ ^T cells could be reversed by a neutralizing antibody against IL-6R.

**Conclusion:**

we showed that enhancement of IL-6R expression on CD4^+ ^T cells upon HBV infection contributes to increased Th17 response in patients with CHB.

## Background

Hepatitis B virus (HBV) infection is a major public health problem worldwide, especially in China, where nearly 7.18% of the population is persistently infected with HBV and most of them develop chronic hepatitis B [1,2]. Previous studies have revealed that cellular immunity is critical for the outcome of HBV infection [3]. While HBV-specific CD4**^+ ^**T cells and CD8^+ ^T cells have been demonstrated to be essential for the control of HBV infection[4-7], antigen non-specific T cells infiltrating the liver are reported to be involved in the live injury[8,9].

Th17 cells are a new lineage of peripheral CD4**^+ ^**T cells which have been identified as a proinflammatory T cell subset [10,11]. In human, IL-6 in combination with TGF-β and IL-1β drive naive CD4**^+ ^**T cell to differentiate into Th17 cells [10,12-14]. Th17 cells can produce multiple cytokines including IL-17A (also known as IL-17), IL-17F, IL-21 and IL-22[12,15]. IL-17, a major effector cytokine of Th17 cells, is involved in mobilizing, recruiting, and activating neutrophils and leads to massive tissue inflammation[16]. Recent studies suggested that Th17 cells also play a central role in the immune-mediated liver injury [17-20]. In particular, Zhang et al reported that antigen non-specific Th17 response was increased in patients with chronic hepatitis B (CHB) and the peripheral Th17 frequency in CHB patients was closely associated with the degree of liver damage which determined by serum alanine amino-transferase (ALT) levels and liver histological activity index(HAI) scores[17]. Consistently, Ge et al demonstrated that the frequency of Th17 cells was positively correlated with serum ALT levels in CHB patients [20]. However, the regulatory mechanism of Th17 responses in patients with HBV infection remains unclear.

In this study, we have found that the increased Th17 response in patients with CHB is correlated with the enhanced IL-6 receptor (IL-6R) expression on CD4^+ ^T cells. In addition, our results indicated that up-regulation of IL-6R expression on CD4^+ ^T cells is important for increased Th17 responses in patients with CHB. These findings suggest that IL-6R can be a novel target for immunotherapy of hepatitis induced by HBV infection.

## Materials and methods

### Subjects

Blood samples were collected from patients with chronic hepatitis B (CHB, n = 40), subjects of asymptomatic HBV carrier (AsC, n = 25), patients with acute hepatitis B (AHB, n = 11). All of them were diagnosed according to the described criteria [17,21,22]. All CHB patients have not received anti-virus treatment (anti-viral drug such as interferon or nucleotide analogues) for at least one year. Individuals with concurrent hepatitis C virus, hepatitis D virus, or hepatitis G virus, or human immunodeficiency virus (HIV) infection, individuals with autoimmune liver diseases, and individuals who met clinical or biological criteria of bacterial or fungal infection were excluded. Twenty-nine age- and sex-matched healthy donors (HD) were enrolled as controls. The study protocol was approved by the ethics committee of Shenzhen Third People's Hospital and written informed consent was obtained from each subject. The basic characteristics of these subjects are listed in Table 1.

**Table 1:** Clinical Characteristics of the Populations Enrolled in the Study

Group	HD^a ^	AsC^b ^	CHB^c ^	AHB^d ^
Case	29	25	40	11
Sex(male/female)	20/9	16/9	28/12	7/4
Age(years)	24.5 ± 7.5	29.3 ± 9.2	30.6 ± 7.7	25.5 ± 4.0
ALT^e ^(U/L)	< 40	21.9 ± 8.4	264.2 ± 171.2	1169.8 ± 617.8
TBIL^f ^(μmol/L)	< 20	13.7 ± 4.2	27.3 ± 19.3	214.7 ± 116.6
HBV DNA (log10 copies/mL)	ND^g ^	3.8 ± 3.6	6.0 ± 1.4	5.1 ± 1.4
HBsAg (+/-)	0/29	25/0	40/0	11/0
HBeAg (+/-)	0/29	13/12	21/19	6/5
HBeAb (+/-)	0/29	12/13	19/21	5/6

Data are expressed as mean ± 1 standard deviation (SD). ^a ^HD, healthy donor; ^b ^AsC, asymptomatic HBV carrier; ^c ^CHB, chronic hepatitis B; ^d ^AHB, acute hepatitis B; ^e ^ALT, alanine amino-transferase; ^f ^TBil, total bilirubin; ^g ^ND, not determined.

### Intracellular staining and flow cytometry analysis

Fluorescence-conjugated antibodies against CD3, CD4, CD8, IFN-γ, IL-17, CD126 (IL-6R) were purchased from BD Biosciences (San Jose, CA). For intracellular staining, fresh heparinized peripheral blood (250 μL) was incubated with phorbol 12-myristate 13-acetate (PMA, 50 ng/ml; Sigma-Aldrich, St. Louis, MO), ionomycin (1 μg/ml; Sigma-Aldrich), and BFA (0.4 μM, BD PharMingen) in 750 μL RPMI 1640 medium supplemented with 10% fetal calf serum (FCS) for 6 hours, 37°C. Cells were stained for cell surface markers first, and then the blood was lysed with fluorescence-activated cell sorting (FACS) lysing solution (BD PharMingen) and further permeabilized, stained with the corresponding intracellular antibody. Flow cytometry analysis was performed on cells acquired using a FACSCalibur (BD Biosciences, San Jose, CA) and data were analyzed using FACSDiva (BD Biosciences)or FlowJo software (Tristar, San Carlos, CA).

### Cell purification

Peripheral blood mononuclear cells (PBMCs) were isolated from fresh whole blood by density gradient centrifugation using Lymphoprep (Axis-Shield, Oslo, Norway). Total CD4^+ ^T cell were purified by positive selection using microbeads according to the manufacturer's instructions (BD Biosciences). The purity of the CD4^+ ^T cell was > 90%. Freshly purified cells were incubated in complete RPMI 1640 medium containing 10% FCS, 2 mM L-glutamine, 100 U/ml penicillin, and 100 μg/ml streptomycin.

### PBMCs culture

To analyze the effect of HBcAg on IL-6R and IL-17 expression by CD4^+ ^T cells, PBMCs were cultured in the absence or presence of HBcAg (10 μg/mL; ARP, Belmont, MA), or LPS(1 μg/ml; Peprotech, Rocky Hill, NJ). At different time points after incubation as indicated in legends, cells were harvested and CD4^+ ^T cells were isolated for quantification of IL-17/IL-6R messenger RNA (mRNA) expression. Aliquot of cultured cells were also subjected flow cytometry analysis of IL-6R expression on CD4^+ ^T cells. Supernatant were collected for cytokines measurement.

### IL-6R blockade treatment

Purified CD4^+ ^T cells from CHB were incubated in medium alone or stimulated with PMA (50 ng/ml; Sigma-Aldrich) and ionomycin (1 μg/ml; Sigma-Aldrich) for 6 h in the presence of 5 μg/ml of brefeldin A(0.4 μM, BD PharMingen) or with HBcAg (10 μg/mL, ARP) for 24 h, neutralizing antibody against IL-6R(R&D systems, Minneapolis, MN) were added at a concentration of 20 μg/ml 30 min before the addition of PMA or HBcAg. Cells were harvested for intracellular cytokine staining and analyzed by flow cytometry. Cell culture supernatants were harvested and the concentration of IL-17, IFN-γ was determined by ELISA.

### RNA extraction and real-time RT-PCR

Total RNA was extracted from purified CD4^+ ^T cells with RNeasy Mini Kit (Qiagen, Santa Clarita, CA) according to the manufacturer's protocol. The RNA was reverse-transcribed to complementary DNA (cDNA) using oligo (dT) primers at 37°C for 15 minutes and at 85°C for 5 seconds. Quantitative real-time PCR was performed using Applied Biosystems 7500 sequence detection system. The primers and probes of IL-17(cat:Hs00936345) and inner control GAPDH(cat:Hs02758991) for real-time PCR were purchased from Applied Biosystems (Foster City, CA). IL-6R primer and probe(Takara, China) sequences were as follows:

forward primer: 5-AAGACAATG CCACTGTTCACTG-3;

reverse primer: 5-GGTAGCATGAATAGTTTCCAGAGTC-3;

probe: 5-FAM-ACCGACCTCAGCAGCAGCCTCCTT-Eclipse-3.

Results are expressed in terms of relative mRNA quantification calculated by using the arithmetic formula 2^-ΔCtΔCt ^[23].

### ELISA assay

The concentrations of IL-17, TGF-β, IL-6, and IL-1β in culture supernatants and TGF-β, IL-6, IL-1β in plasma were determined by ELISA (R&D Systems, Minneapolis, MN) following manufacturer's guidelines.

### Statistical Analysis

All statistical tests were performed with SPSS 13.0(Chicago, IL) or Prism 3.0 (GraphPad, La Jolla, CA). The one-way analysis of variance/Newman-Keuls multiple comparison test was used for statistical analysis to compare the differences among multiple groups. The unpaired *t *test was used to analyze the difference between two groups. The Wilcoxon matched pair t test was used to analyze the effect of antigens on IL-6R and IL-17 expression by CD4^+ ^T cells *in vitro*. Correlation analysis was performed by Pearson's *t *test. For all test, two-sided P < 0.05 was considered statistically significant.

## Results

### Increased Th17 responses were associated with liver injury in patients with HBV infection

**Figure 1:**
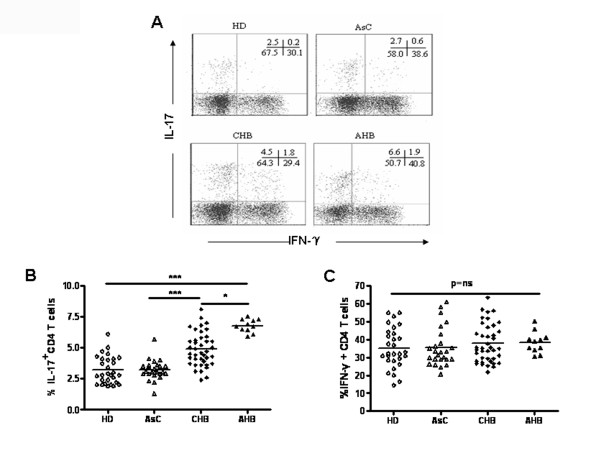
**Th17 cells were increased in peripheral blood of CHB and AHB patients**. (A) Dot plots show intracellular staining for IL-17-producing CD4 T cells (Th17) and IFN-γ-producing CD4 T cells (Th1) in the peripheral blood of HD, AsC, CHB and AHB. Pooled data indicates the percentage of (B) Th17 and (C) Th1 within CD4 T cells in peripheral blood of HD (n = 29), AsC (n = 25), CHB (n = 40) and AHB (n = 11) patients. *p < 0.05; ***p < 0.001; Horizontal bars represent the median values; ns, not significance.

Previous studies within CHB patients suggested that Th17 cells play an important role in HBV-induced inflammatory responses [17-20]. To better understand the role of Th17 cells in HBV infection, we extended these findings by comparing Th17 responses in patients with acute hepatitis B (AHB), asymptomatic HBV carrier (AsC), as well as patients with CHB. Consistent with previous reports, we found that Th17 responses were significantly increased in patients with CHB in comparison to healthy donors. Moreover, among HBV infected individuals, Th17 responses were significantly higher in AHB patients than CHB patients, and both populations have significantly higher Th17 responses than subjects with AsC (P < 0.001, Figure 1A and 1B). In contrast, there was no difference in Th17 responses between AsC and HD. Furthermore, there were no differences in Th1 responses (the frequency of IFN-γ producing CD4 ^+ ^T cells) among different populations (P = ns, Figure 1C). Therefore, our data confirmed that Th17 responses correlated with liver injury in HBV infected individuals.

### Cytokines related to Th17 differentiation were increased in HBV infected subjects

**Figure 2:**
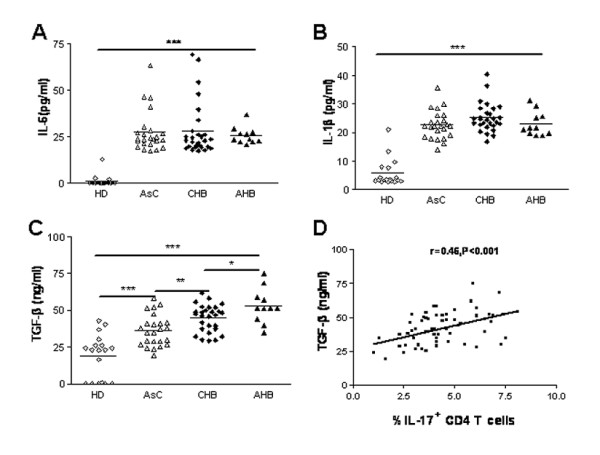
**The plasma concentrations of IL-6, IL-1β, TGF-β were increased in HBV infected patients**. The concentrations of (A) IL-6, (B) IL-1β, (C)TGF-β in plasma of HD (n = 18), AsC (n = 24), CHB (n = 28), AHB (n = 11). (D) Correlation analysis between the plasma concentration of TGF-β and the frequency of Th17 in peripheral blood of HBV infected individuals (n = 63). *p < 0.05; **p < 0.01; ***p < 0.005. Horizontal bars represent the median values of indicated index; Solid line, linear growth trend; r, correlation coefficient. P-values are shown.

Previous studies suggested that TGF-β or IL-1β facilitates Th17 cells development from naïve CD4T cells in the presence of IL-6 [10,12-14]. To investigate whether increased Th17 responses observed in patients with CHB and AHB were due to an altered cytokine environment induced upon HBV infection, we examined plasma concentrations of TGF-β, IL-1β, and IL-6 in different populations. Consistent with previous reports [17,18], we found that concentrations of plasma IL-6, IL-1β, and TGF-β were significantly higher in patients with CHB and AHB than those in HD (P < 0.001, Figure 2A, B, C). However, the concentrations of plasma IL-6 and IL-1β in ASC were not different from those of HBV infected individuals with hepatitis (CHB &AHB). In contrast, the concentration of plasma TGF-β was significantly different among all four groups. In addition, correlation analysis showed that the concentration of plasma TGF-β, but not IL-6, IL-1β, significantly correlated with the frequency of Th17 cells in periphery blood within HBV infected subjects (r = 0.46, P < 0.001, Figure 2D). But in CHB patients, TGF-β, IL-6 and IL-1β, all did not correlated with the frequency of periphery Th17 cells (data not shown). Thus, further experiments are warranted to clarify the exact role of increased TGF-β, IL-6 and IL-1β in regulating Th17 development upon HBV infection.

### Elevated IL-6R expression on CD4 ^+ ^T cells correlated with an increased Th17 response in HBV infected individuals

**Figure 3:**
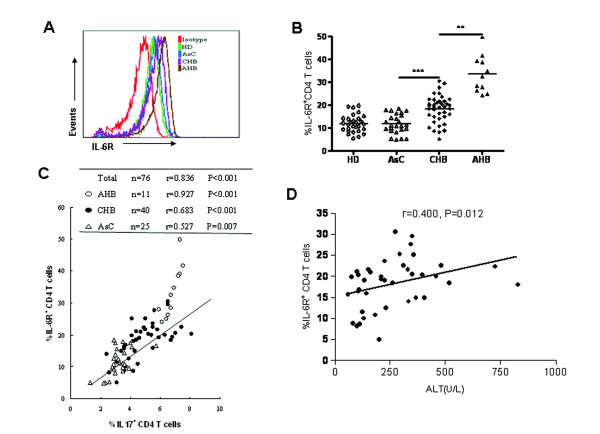
**Increased expression of IL-6R on CD4 T cells in CHB and AHB patients**. (A) Flow cytometric analysis of membrane IL-6R expression on CD4 T cells of HD, AsC, CHB and AHB. Representative histograms of IL-6R expression on CD4 T cells of each population were shown. (B) Pooled data indicate the proportion of IL-6R expression on CD4 T cells in HD (n = 29), AsC (n = 25), CHB (n = 40), AHB (n = 11). (C) Correlation analysis between the frequency of Th17 cells and the proportion of IL-6R expressing on CD4 T cells in HBV infected patients (n = 76), inserted table indicated the correlation coefficient of two parameters within AHB, CHB and AsC. (D) Correlation analysis between ALT and the proportion of IL-6R expressing on CD4 T cells in CHB patients. *, p < 0.05; **, p < 0.01; ***, p < 0.001; horizontal bars represented the median values; solid line, linear growth trend; r, correlation coefficient. P-values are shown.

Although previous studies showed that IL-6 is critical for the differentiation of Th17 cells from naïve T cells [14], the plasma concentration of IL-6 in HBV infected individuals was not correlated with the level of Th17 responses. Because IL-6 receptor (IL-6R) is mandatory for the bioactivity of IL-6 and we previously reported that down-regulation of IL-6R expression on CD4^+ ^T cells by *Mycobacterium *tuberculosis is an important mechanism underlying reduced Th17 responses in patients with tuberculosis [24-26], we decided to investigate whether IL-6R expression is also correlated with Th17 responses in CHB patients. Our result showed that the percentage of IL-6R expressing CD4^+ ^T cells in peripheral blood of AsC was not different from that of HD. In contrast, both AHB and CHB patients had significantly higher percentage of IL-6R expressing CD4^+ ^T cells than AsC patients. The percentage of IL-6R expressing CD4^+ ^T cells in AHB patients was also significantly higher than that of CHB patients (Figure 3A, B). Correlation analysis demonstrated that the percentage of IL-6R expressing CD4^+ ^T cells was significantly correlated with the frequency of Th17 cells within all HBV infected subjects (r = 0.836, P < 0.001, Figure 3C), or within patients with AHB (r = 0.927, P < 0.001), or CHB(r = 0.683, P < 0.001), or AsC(r = 0.527, P = 0.007). Furthermore, the percentage of IL-6R expressing CD4^+ ^T cells was also significantly correlated with serum ALT levels in CHB patients (r = 0.400, P = 0.012, Figure 3D). These findings suggested that alteration of IL-6R expression on CD4^+ ^T cells upon HBV infection might account for increased Th17 response as well as elevated ALT level in patients with hepatitis B.

### HBcAg enhanced IL-17 production and IL-6R expression by CD4 ^+ ^T cells from patients with chronic hepatitis B in vitro

**Figure 4:**
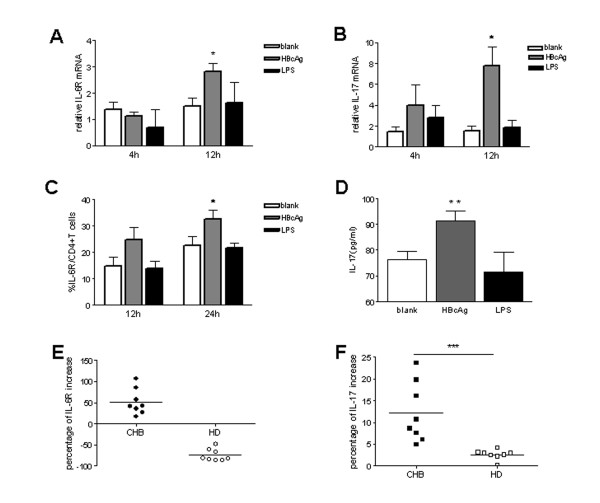
**HBcAg up-regulate IL-17 and IL-6R expression by CD4 T cells *in vitro***. PBMCs from CHB patients (n = 8) were cultured in medium alone, or in the presence of HBcAg (10 μg/ml) or LPS (1 μg/ml). Relative IL-6R (A) and IL-17(B) mRNA expression of CD4 T cells purified from cultured cells harvested at indicated time after incubation were determined by real-time RT-PCR; (C) the percentage of IL-6R expression on CD4 T cells in cultured cells collected at indicated time after incubation were determined by flow cytometry; (D) IL-17 production in culture supernatants harvested at 48 hours after incubation was measured by ELISA. (E) Modulation of IL-6R expression on CD4 T cells after incubation with HBcAg for 24 hours and (F) IL-17 production after incubation with HBcAg for 48 hours in CHB patients (n = 8) and HD (n = 8). The percentage of increase of IL-6R on CD4 T cells was calculated by dividing the frequencies of IL-6R expressing CD4 cells determined by flow cytometry cultured in medium alone with those cultured in the presence of HBcAg. Similar formulation was used to calculate the percentage of increase of IL-17 production measured by ELISA in culture supernatant. *, P < 0.05; **, p < 0.01, ***, P < 0.001; the bars represent means with standard deviations. Horizontal bars represent the median values.

The finding that increased Th17 responses were correlated with enhanced IL-6R expression on CD4 ^+ ^T cells in CHB patients prompted us to hypothesize that HBV and/or its products could modulate Th17 responses through regulating IL-6R expression on CD4 ^+ ^T cells. To test this hypothesis, we investigated the effect of HBV core antigen on modulating IL-6R expression and IL-17 production by CD4 ^+ ^T cells *in vitro*. As expected, incubation of PBMCs from CHB patients in the presence of HBcAg not only resulted in significantly enhanced IL-6R expression by CD4 ^+ ^T cells at both mRNA (Figure 4A) and protein level (Figure 4C), but also increased IL-17 mRNA level by CD4 ^+ ^T cells (Figure 4B) and elevated IL-17 production in supernatant of PBMCs culture (Figure 4D). In comparison, no significant enhancement effect on IL-6R and IL-17 expression was observed when PBMCs were incubated with LPS. Kinetics analysis showed the effect of HBcAg on enhancing IL-17 and IL-6R mRNA expression could be observed as early as 4 hours and peaked at 12 hours after treatment (Figure 4A and 4B).

To determine whether *in vivo *chronic exposure to HBV infection influences the capability of CD4^+ ^T cells to express IL-6R and IL-17 in response to HBcAg stimulation *in vitro*, we stimulated CD4^+ ^T cells from CHB patients with HBcAg and compared their responsiveness with those from HD individuals. We found that, upon HBcAg stimulation, IL-6R expression on CD4^+ ^T cells from CHB patients was significantly increased. In contrast, IL-6R expression on CD4^+ ^T cells from HD was decreased upon HBcAg stimulation (Figure 4E). Consistently, we found that PBMCs from CHB patients up-regulated IL-17 production upon HBcAg stimulation to a higher percentage than those from HD (P < 0.001, Figure 4F).

Taken together, these data suggested that modulation of IL-6R expression on CD4^+ ^T cells is an important mechanism underlies the enhanced Th17 responses in patients with chronic hepatitis B.

### An anti-IL-6R antibody inhibited PMA/ionomycin and HBcAg induced IL-17 production by CD4 ^+ ^T cells

**Figure 5:**
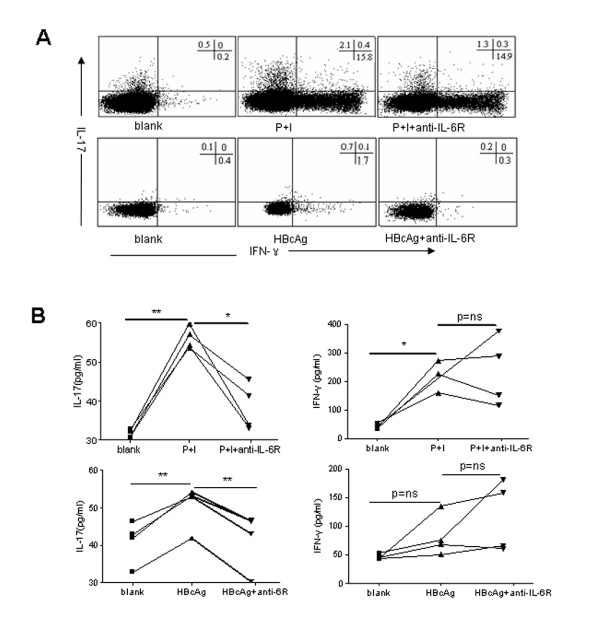
**Blockade of IL-6R signaling inhibited PMA/ionomycin and HBcAg induced IL-17 production by CD4 T cell**. Purified CD4 T cells from CHB patients (n = 4) were cultured in medium alone (blank), in the presence of PMA (50 ng/ml) and ionomycin (1 μg/ml) for 6 h (P+I), or in the presence of HBcAg (10 μg/mL) for 24 h, without or with neutralizing antibody against IL-6R (anti-IL-6R, 20 μg/ml). Cells and supernatant were harvested at 6 hours for P+I stimulation, and 24 hours for HBcAg stimulation, with or without anti-IL-6R. (A) The frequency of IFN-γ- and IL-17- producing CD4 T cells were determined by flow cytometry analysis. Representative dot plots showed IFN-γ- and IL-17- expressing cells within gated CD3CD4 T cells. (B) IFN-γ and IL-17 production released in supernatant of CD4 T cells culture was measured by ELISA. *, P < 0.05; **, p < 0.01, ns, not significant.

To validate whether enhanced IL-6R expression on CD4^+ ^T cells is causative of elevated Th17 response in patients with chronic hepatitis B, we studied the effect of blockade of IL-6R signaling on PMA/ionomycin and HBcAg induced IL-17 production by purified CD4T cells *in vitro*. Blockade of IL-6R signaling by a neutralizing antibody against IL-6R significantly inhibited PMA/ionomycin-stimulated IL-17 expression by CD4^+ ^T cells, as demonstrated by reduced frequency of IL-17 producing CD4^+ ^T cells (Figure 5A) and decreased IL-17 secreted in supernatant of purified CD4^+ ^T cell culture (Figure 5B). Similarly, treatment with anti-IL-6R significantly inhibited HBcAg-induced IL-17 production by CD4^+ ^T cells. Moreover, no effect of IL-6R blocking with anti-IL-6R on IFN-γ production by CD4^+ ^T cells has been observed (Figure 5A and 5B). Thus, these data confirmed that enhancement of IL-6R expression on CD4^+ ^T cells accounted, at least partially, for the elevated Th17 responses in patients with chronic hepatitis B.

### HBcAg and LPS Induced Th17 differentiation related cytokines production by PBMCs in vitro

**Figure 6:**
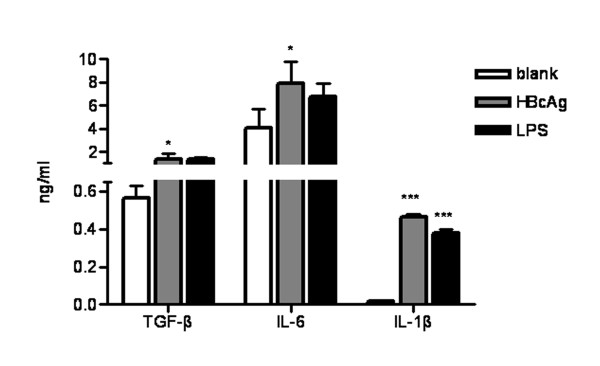
**HBcAg induced TGF-β, IL-6 and IL-1β production by PBMCs *in vitro***. PBMCs from CHB patients (n = 9) were cultured in the presence of HBcAg (10 μg/ml) or LPS (1 μg/ml) medium alone. After 48 h, culture supernatant was collected and the concentrations of TGF-β, IL-6 and IL-1β were measured by ELISA. *, P < 0.05. ***, P < 0.001.

The finding that elevated plasma TGF-β, IL-1β, and IL-6 in HBV infected individuals suggested that HBV may have direct effect in inducing these cytokines production. To test this hypothesis, we investigate the effect of HBcAg on modulating cytokine production by PBMCs. We found both PBMCs (Figure 6) secreted significantly more TGF-β, IL-6 and IL-1β upon HBcAg stimulation than cultured with medium alone. Notably, similar effects were also observed in PBMCs culture in the presence of LPS. Since variety of pathogens contains LPS, our data suggested that both HBV and/or bacterial pathogens superinfected post onset of HBV infection could induce cytokines milieu that facilitates the differentiation of Th17 response.

## Discussion

Recent studies have shown that Th17 responses were significantly increased in patients with CHB. Correlation analysis also suggested that Th17 cells play an important role in inflammatory response and cell mediated liver injury of CHB patients [17-20]. In this study, we extended these observations by finding that Th17 responses were even significantly higher in AHB patients than CHB patients. In contrast, Th17 responses were not different between asymptomatic HBV carriers and healthy donors. Taken together, these data provided substantial evidences that Th17 cells contribute to inflammatory responses and cell-mediated liver injuries in individuals with HBV infection. Accordingly, understanding the regulatory mechanism of Th17 responses in HBV infected individuals is of particular importance, considering it may provide novel strategies for the treatment of patients with hepatitis B.

In attempt to investigate the mechanism underlying the increased Th17 response in patients with CHB, we first looked at the cytokine milieu related to the Th17 differentiation and found that TGF-β, IL-6 and IL-1β were increased in plasma of HBV infected individuals, irrespective their clinical manifestation. Consistent with these findings, *in vitro *experiments showed that PBMCs produced TGF-β, IL-1β, and IL-6 upon stimulation with HBcAg. However, correlation analysis indicated that only the concentration of plasma TGF-β, but not IL-6 nor IL-1β, significantly correlated with increased Th17 responses in HBV infected patients. Thus, unlike the inhibitory role TGF-β plays during the HCV-specific Th17 response [27], TGF-β may facilitate the development of Th17 responses in HBV infected subjects. Nevertheless, because TGF-β is also involved in CD4^+ ^CD25^+ ^regulatory T cells (Treg) differentiation and our previous study have found that the concentration of plasma TGF-β in CHB patients also correlated with the frequency of Treg [28], the exact role of TGF-β in regulating Th17 response as well as balancing between Th17 and Treg in HBV infected individuals remains to be elucidated.

Consistent with our previous finding in patients with tuberculosis, we found IL-6R expression on CD4^+ ^T cells, but not plasma IL-6, correlated with the Th17 response in HBV infected individuals [29]. However, unlike *Mycobacterium *tuberculosis antigens which down-regulated IL-6R expression on CD4**^+ ^**T cells from patients with tuberculosis, HBV antigen (HBcAg) up-regulated IL-6R expression on CD4^+ ^T cells from patients with CHB. More importantly, our *in vitro *data validated that blockade of IL-6R signaling on CD4^+ ^T cells significantly inhibited antigen non-specific (PMA/Ionomycin-stimulated) and HBcAg antigen-specific IL-17, but not IFN-γ, production by CD4^+ ^T cells, which was not surprised considering the fact that no requirement of IL-6 for Th1 differentiation. Thus, while both infections modulate IL-6R expression on CD4^+ ^T cells, the effect of *Mycobacterium *tuberculosis infection was markedly different from that of HBV infection. In addition, the difference was likely due to *in vivo *"tuning" of CD4^+ ^T cells by chronic exposure to inflammatory environment superimposed by HBV infection (including circulating HBV antigens), since HBcAg, similar to *Mycobacterium *tuberculosis antigens, down-regulated IL-6R expression on CD4^+ ^T cells in healthy donors. While further investigations are warranted to identify the exact mechanisms that account for "tuning" CD4^+ ^T cells *in vivo*, circulating HBV antigens as well as the cytokines induced by HBV infection might be most potential candidates that are responsible for such different effect. For example, elevated TGF-β in patients with hepatitis B might "tune" CD4^+ ^T cells with increased sensitivity to IL-6R signaling through inhibition of SOCS3[30,31].

## Conclusions

In summary, our present study provided substantial evidences that enhancement of IL-6R expression on CD4^+ ^T cells by HBV is an important mechanism for increased Th17 responses in patients with CHB. First, the percentage of IL-6R expression on CD4^+ ^T cells correlated with the frequency of Th17 cells in peripheral blood *in vivo*. Second, HBcAg up-regulated IL-6R and IL-17 expression by CD4^+ ^T cells from CHB patients *in vitro*. Third, the blockade of IL-6R signaling reversed the increase of IL-17 production by CD4^+ ^T cells. Combined with previous reports about the usefulness of neutralizing anti-IL-6R monoclonal antibody to treat autoimmune diseases via inhibiting Th17 responses[32-34], our finding suggests a novel strategy for treating CHB patients, e.g. inhibiting IL-6R expression by anti-IL-6R antibodies to restrain Th17-mediated liver damage.

## List of Abbreviations

Th17: Interleukin-17-producing CD4^+ ^T cell; HBV: hepatitis B virus; CHB, chronic hepatitis B; ALT: alanine amino-transferase; TBil: total bilirubin; HAI: histological activity index; HBcAg: Hepatitis B core antigen; HBsAg: Hepatitis B surface antigen; HIV: human immunodeficiency virus; AsC: asymptomatic HBV carrier; AHB: acute hepatitis B; HD: health donor; PBMCs: Peripheral blood mononuclear cells; mRNA: messenger RNA.

## Competing interests

The authors declare that they have no competing interests.

## Authors' contributions

BZ, XC, GY, ZG, co-defined the research theme. XC and FZ co-designed methods and experiments. FZ, MZ carried out the laboratory experiments, analyzed the data and drafted the manuscript., SY, JY, QH, HL co-worked on the associated data collection and their interpretation. BZ, XC and BL revised the paper critically for important intellectual content. All authors have seen and approved the manuscript.
